# *Fmr1*-Deficiency Impacts Body Composition, Skeleton, and Bone Microstructure in a Mouse Model of Fragile X Syndrome

**DOI:** 10.3389/fendo.2019.00678

**Published:** 2019-10-02

**Authors:** Antoine Leboucher, Patricia Bermudez-Martin, Xavier Mouska, Ez-Zoubir Amri, Didier F. Pisani, Laetitia Davidovic

**Affiliations:** ^1^Université Côte d'Azur, CNRS, IPMC, Valbonne, France; ^2^Université Côte d'Azur, CNRS, Inserm, iBV, Nice, France; ^3^Université Côte d'Azur, CNRS, LP2M, Nice, France

**Keywords:** fragile X syndrome, bone microstructure, skeleton, tomography, trabecula, muscle, physical activity

## Abstract

Fragile X syndrome (FXS) is a neurodevelopmental disorder associated with intellectual disability, hyperactivity, and autism. FXS is due to the silencing of the X-linked *FMR1* gene. Murine models of FXS, knock-out (KO) for the murine homolog *Fmr1*, have been generated, exhibiting CNS-related behavioral, and neuronal anomalies reminiscent of the human phenotypes. As a reflection of the almost ubiquitous expression of the *FMR1* gene, FXS is also accompanied by physical abnormalities. This suggests that the *FMR1-*deficiency could impact skeletal ontogenesis. In the present study, we highlight that *Fmr1-*KO mice display changes in body composition with an increase in body weight, likely due to both increase of skeleton length and muscular mass along with reduced visceral adiposity. We also show that, while *Fmr1-*deficiency has no overt impact on cortical bone mineral density (BMD), cortical thickness was increased, and cortical eccentricity was decreased in the femurs from *Fmr1-*KO mice as compared to controls. Also, trabecular pore volume was reduced and trabecular thickness distribution was shifted toward higher ranges in *Fmr1-*KO femurs. Finally, we show that *Fmr1*-KO mice display increased physical activity. Although the precise molecular signaling mechanism that produces these skeletal and bone microstructure changes remains to be determined, our study warrants further investigation on the impact of *FMR1*-deficiency on whole-body composition, as well as skeletal and bone architecture.

## Introduction

Fragile X syndrome (FXS) is a neurodevelopmental disorder associated with intellectual disability, attention deficits, and hyperactivity disorders, and autism spectrum disorders (1, 2). FXS affects 1/4,000 males and 1/7,000 females worldwide and is caused by the silencing of the X-linked Fragile X Mental Retardation 1 (*FMR1*) gene located in a fragile chromosomal site in 27q3 (3, 4). In FXS patients, abnormal expansions of CGG trinucleotide repeats in the 5′ untranslated region of the *FMR1* gene lead to an hypermethylation of the upstream CpG island in the gene promotor and to *FMR1* silencing (3–5). Murine *Fmr1*-knock-out (KO) models of FXS have been generated, exhibiting CNS-related behavioral, and neuronal anomalies reminiscent of the human phenotypes (6, 7). The *FMR1* gene is not only expressed in the central nervous system (CNS) but also in a variety of peripheral tissues, including adipose tissue, and liver (8–11). Adult skeletal and cardiac muscle are the only peripheral tissues tested so far not expressing FMRP, while both white adipose tissue (WAT) and brown adipose tissue (BAT) express FMRP (8–12). This suggests that long-term pathological consequences of *FMR1*-deficiency are not solely confined to the central nervous system disorders and more likely extend to physiological dysfunctions in peripheral systems. We have previously demonstrated in FXS mouse model how *Fmr1-*deficiency increased systemic utilization of lipids, reduced adiposity and provoked significant changes in metabolic homeostasis, some of which were also translatable to FXS patients (11). Besides presenting metabolic phenotypes, FXS is also accompanied by physical abnormalities (13). In subsets of FXS patients, morphometric studies have highlighted increased stature and height (14) and a general overgrowth in prepubertal boys affected by FXS (13, 15). FXS patients present connective tissue dysplasia (16), dental and mandibular anomalies (17), orthopedic anomalies such as scoliosis (18), and abnormal metacarpophalangeal pattern profile (19). In addition, FXS patients display specific craniofacial abnormalities with reduced facial depth, hypoplasticity of the nasal bone–cartilage interface and narrow mid-facial width exaggerating ear prominence (20). This suggests that *Fmr1-*deficiency could have widespread peripheral effects, including on skeletal ontogenesis. Besides subtle craniofacial anomalies with morphometric changes in the mandible and skull (20), skeletal particularities, and bone microstructure have not yet been studied in FXS mouse model. In this study, we examined in details body composition and bone microstructure of adult *Fmr1-*KO male mice.

## Materials and Methods

### Animals Procedures

*Fmr1-*KO2 mice on the C57Bl/6J background used in this study were originally described in Mientjes et al. (7) and were named *Fmr1-*KO all throughout the manuscript. *Fmr1-*heterozygous females and *Fmr1-*KO male founders were obtained from Rob Willemsem (Erasmus University, Rotterdam, Netherlands) and backcrossed for at least 10 generations on a C57Bl6/J WT background (Janvier Labs, France). All *Fmr1-*WT and *Fmr1*-KO male used in this study were littermates born to *Fmr1* heterozygous females mated with *Fmr1*-KO males. Pups were weaned at 28 days of age, identified by ear tags, and genotyped by PCR as described (7). Pups from various litters were then randomly grouped according to their genotype in cages and had *ad libitum* access to water and standard chow (reference 4RF25, Mucedola) composed of cereals (53.7%), animal proteins (4.7%), vegetal proteins (30.5%), lipids (soy oil, 1.4%), vitamins and minerals (4.1%), amino acids (0.1%). Animals were housed in a temperature (22–24°C) and hygrometry (70–80%)- controlled room with a 12 h light-dark cycle (lights on at 08:00). Animals were housed in medium-size (up to 5 animals/cage) or large cages (up to 10 animals/cage) filled with wooden bedding, one plastic house and nesting material for enrichment. All the described experiments were performed on male animals at exactly 4-months (16 weeks) of age. After cervical dislocation, muscle *Tibialis anterior*, visceral, and subcutaneous WAT, as well as interscapular BAT were dissected by trained personnel and weighed with a precision balance.

### Osteocalcin Measure

Animals were anesthetized and blood was withdrawn by cardiac puncture, incubated for at least 30 min at room temperature (RT) then centrifuged at 400 g, 10 min, RT. Serum was collected and immediately snapped-frozen in liquid nitrogen prior storage at −80°C until use. Osteocalcin was measured in serum using a dedicated ELISA kit (Takara), according strictly to the manufacturer's instructions.

### Microcomputerised Tomography Analysis: Skeleton Morphology

**Figure 1:**
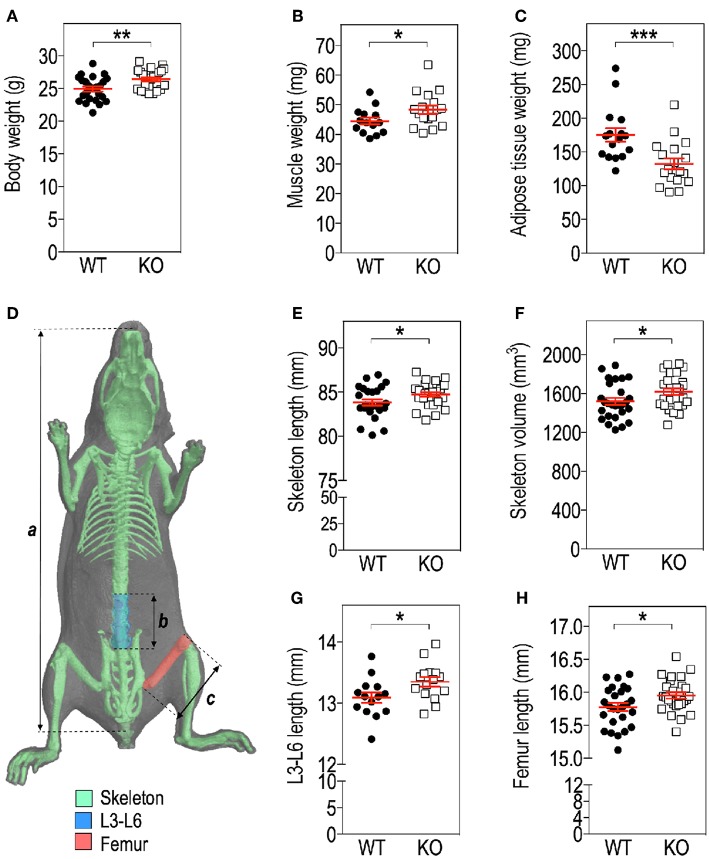
*Fmr1*-deficiency modifies body mass distribution. Body weight **(A)**, *Tibialis anterior* muscle **(B)**, as well as visceral white adipose tissue (WAT) **(C)** weights in *Fmr1*-KO and WT 4-months old male mice. **(D)** Anatomical coordinates used for analysis of: *a*, skeleton length; *b*, vertebral L3-L6 length; *c*, maximal femur length. Skeleton length **(E)**, skeleton volume **(F)**, vertebral L3-L6 length **(G)**, and femoral length **(H)** were measured in 3D reconstructions of skeleton following X-ray tomography. Data are presented as dot-plots featuring means ± SEM in red. Statistical significance of differences was measured using 2-tailed Student's *T*-test. *p < 0.05; **p < 0.01; ***p < 0.001. For **(A,E,F,H)**, *n* = 24–27 animals/group; for **(B,C,G)**, *n* = 15–18 animals/group.

For the analysis of whole-body bone morphology (Figure 1), anesthetised animals were introduced in a SkyScan μCT −1178 X-ray tomograph (Bruker) and analyzed as previously described (11, 21). Mice were scanned using the following parameters: 104 μm pixel size, 49 kV, 0.5 mm thick aluminum filter and a rotation step of 0.9°. 3D reconstructions and analysis of skeleton, vertebra and femur lengths as well as skeleton volume were performed using NRecon and CTAn software (Skyscan). Skeleton volume was calculated using CTan software by extracting from μCT scans all voxels exceeding a single global CT threshold value, ensuring that differences between study groups are due to experimental effects rather than image-processing effects. The representative skeleton reconstruction from one *Fmr1-*WT animal (Figure 1D) was obtained by further processing of the 3D reconstructed sections using the ImageJ plugin 3D viewer and the BoneJ plugin (22). Bone and skeleton lengths were calculated using the 3D coordinates of the structure extremities specified in Figure 1D. For skeleton length we used the anterior coordinate of the nasal bone and the posterior coordinate of the first caudal vertebra. For femur length, we used the extreme coordinate of the femoral epiphysis and the extreme coordinate of the proximal epiphysis (measured on both femurs and averaged). For L3-L6 vertebra length we used the anterior extreme coordinate of L3 vertebra and the posterior extreme coordinate of L6 vertebra.

### Microcomputerised Tomography Analysis: Bone Microstructure

For measurements of bone mineral density (BMD) and microstructure, we proceeded according to the guidelines for Assessment of Bone Microstructure in Rodents Using Micro–Computed Tomography (23). After necropsy, femora were individually scanned with a SkyScan μCT-1173 X-ray tomograph (Bruker) set with the following parameters: 7.76 μm pixel size, 45 kV, 1 mm thick aluminum filter, 0.5° of rotation step, as previously described (21). 3D reconstructions and bone microstructure analysis were performed using NRecon and CTAn software (Skyscan). Bone microstructure was reconstructed using CTan software by extracting from μCT scans all voxels exceeding a single global CT value.

The region of interest (ROI) for calculations of femur cortical bone microstructure parameters was defined as a mid-shaft segment of 400 μm starting at 55% of the total femur length from the proximal epiphysis extremity (corresponding on average to a distance of 8.68 mm for WT animals and 8.77 mm for KO animals) and extending up to 400 μm distally. The ROI for calculations of femur trabecular bone microstructure parameters was defined as a 900 μm-segment of the distal metaphysis, located on average 450 μm away from the epiphyseal line and excluding cortical bone. The segment started at a distance equivalent to 13.5% of the total femur length measured from the distal extremity of the femur, corresponding on average to a distance of 2.13 mm in the WT and 2.15 mm in the KO.

For volumetric cortical BMD calculation, a BMD phantom rod pair supplied by the manufacturer and suitable for mouse bone analysis (2 mm of diameter) with different known densities (0.25 and 0.75 g.cm^−3^ calcium hydroxyapatite) was scanned along with the samples, using scan settings identical to those for acquisitions on femurs. Individual cortical (total cross-sectional object area, object area per slice, cortical area fraction, cortical thickness, mean polar moment of inertia (MMI), eccentricity) and trabecular (bone volume fraction, trabecular number, trabecular thickness, trabecular separation, total volume of trabecular pores) bone microstructure parameters were extracted on each section of the corresponding 3D ROI, using the CTAn software and then averaged as recommended in Bouxsein et al. (23). Their means and standard deviations (SD) for each group are reported in Tables 1, 2. Representative cortical and trabecular ROI from one *Fmr1-*WT and one *Fmr1*-KO animal were obtained by further processing of the 3D reconstructed segments using the ImageJ plugin 3D viewer and the BoneJ plugin (22).

**Table 1:** Comparison analysis of variables for cortical bone morphology in *Fmr1-*KO and WT femurs.

**Abbreviation**	**Variable description**	**Unit**	**Mean WT**	**SD WT**	**Mean KO**	**SD KO**	***p*-value**
vBMD	Volumetric bone mineral density	mg/cm^3^	1.2310	0.0593	1.2190	0.0334	0.5403^a^
Tt.Ar	Mean total cross-sectional object area	mm^2^	0.9402	0.1002	1.0010	0.1179	0.1548^a^
Ct.Ar	Average object area per slice	mm^2^	0.4788	0.0729	0.3885	0.1812	0.1214^a^
Ct.Ar/Tt.Ar	Cortical area fraction	%	53.62	12.28	40.31	20.61	0.0574^a^
Ct.Th	Average cortical thickness	mm	0.1748	0.0106	0.1856	0.0098	**0.0128^a^**
MMI	Mean polar moment of inertia	mm^4^	0.526	0.102	0.544	0.113	0.6613^a^
Ecc	Eccentricity	A. U.	0.7056	0.0717	0.6338	0.0680	**0.0148^b^**

Mean and standard deviations (SD) to the mean are reported for Fmr1-WT and KO animals, as well as p-value for the Student's T-test

(a) or the Mann & Whitney U-test

(b). *Significant p-values (p < 0.05) are bolded. n = 13–15 animals/group*.

**Table 2:** Comparison analysis of variables for trabecular bone microarchitecture in *Fmr1-*KO and WT femurs.

**Abbreviation**	**Variable description**	**Unit**	**Mean WT**	**SD WT**	**Mean KO**	**SD KO**	***p*-value**
BV/TV	Bone volume fraction	%	18.6300	5.1660	22.3700	5.5000	**0.0469^b^**
Tb.N	Trabecular number	nb/mm	1.1110	0.5512	1.1160	0.9092	0.5164^b^
Tb.Th	Trabecular thickness	mm	0.06106	0.007229	0.06624	0.00632	0.0619^a^
Tb.Sp	Trabecular separation	mm	0.2140	0.0257	0.2005	0.0292	0.2295^a^
Tb.Po	Total volume of trabecular pores	mm^3^	1.7320	0.1008	1.5510	0.1443	**0.0007^a^**

Mean and standard deviations (SD) are reported for Fmr1-WT and KO animals, as well as p-value for the Student's T-test

(a) or the Mann & Whitney U-test

(b). *Significant p-values (p < 0.05) are bolded. n = 12–15 animals/group*.

### Locomotor Activity Recordings

Gross locomotor activity was determined in an open field arena. Animals were placed in a brightly illuminated arena (200 Lux) and their movements were recorded for 10 min. An automated tracking system (Anymaze) was used to determine total distance moved, rotations number and number of center entries. The measurement of individual spontaneous activity was made using actimetry chambers (Imetronic) consisting of cages equipped with infrared beams able to detect in real time horizontal and vertical movements (rearings). Animals were individually placed in actimetry chambers under a 12-h light/dark cycle, with free access to food and drinking water. To avoid biases in measurements due to stress possibly inducing hyperlocomotion in a novel environment, an habituation period (Day 0: 11:00 p.m. to Day 1: 8:00 a.m.) preceded the 24 h-recording of horizontal and vertical activity (Day 1: 8:00 a.m. to Day 2: 8:00 a.m.).

### Statistics

Normality of data was assessed using Kolmogorov-Smirnov's test and outliers removed using the robust regression and outlier removal (ROUT) method (24). To compare 2 groups, 2-tailed unpaired Student's *T*-test was used. For non-normal data, raw data were log-transformed to meet normality criteria prior to Student's *T*-test. If normality was not reached after log-transformation, data were analyzed using Mann & Whitney's non-parametrical U-test. Multiple group comparisons were performed using 2-way ANOVA, followed by *post-hoc* tests as indicated in figures legends. Statistical significance was set according to a two-tailed *p*-value (*p*) < 0.05. Statistical analysis was performed using GraphPad Prism version 6.00 for iOS (GraphPad Software, USA).

## Results

### *Fmr1*-Deficiency Impacts Skeleton and Body Mass Distribution

We assessed several basic parameters in 4-months old *Fmr1-*KO and WT male littermates (Figure 1). *Fmr1*-KO mice display a significant increase in total body weight as compared to WT (Figure 1A). Furthermore, we weighed *Tibialis anterior* muscle and epididymal fat depots and showed that muscle weight was significantly increased in *Fmr1*-KO mice, to the expense of visceral adipose tissue that was reduced (Figures 1B,C). In contrast, subcutaneous or BAT weights were similar in *Fmr1-*WT and KO animals (Supplementary Figure 1). This agreed with the reduction of visceral adipose tissue volume and lack of variation in subcutaneous adipose tissue volume observed in a previous study in *Fmr1-*KO animals (11).

To precisely study the skeleton, we measured a number of skeletal parameters on 3D reconstructions of skeleton from micro-computerized X-ray tomography data (Figure 1D). We found that *Fmr1*-deficiency is accompanied by a significant increase in total mouse length, as well as in skeletal volume (Figures 1E,F). Femur maximal length and L3-L6 vertebral length, which are reliable indicators of general skeleton length, were also both significantly increased in *Fmr1-*deficient animals (Figures 1G,H).

Finally, the ratios: muscle weight/body weight, femur length/body length, L4-L6 length/body length were not significantly different between *Fmr1*-KO and WT animals (Supplementary Table 1), indicating that *Fmr1*-KO animals exhibited similar proportions to WT animals.

### *Fmr1*-Deficiency Determines Changes in Bone Microstructure

**Figure 2:**
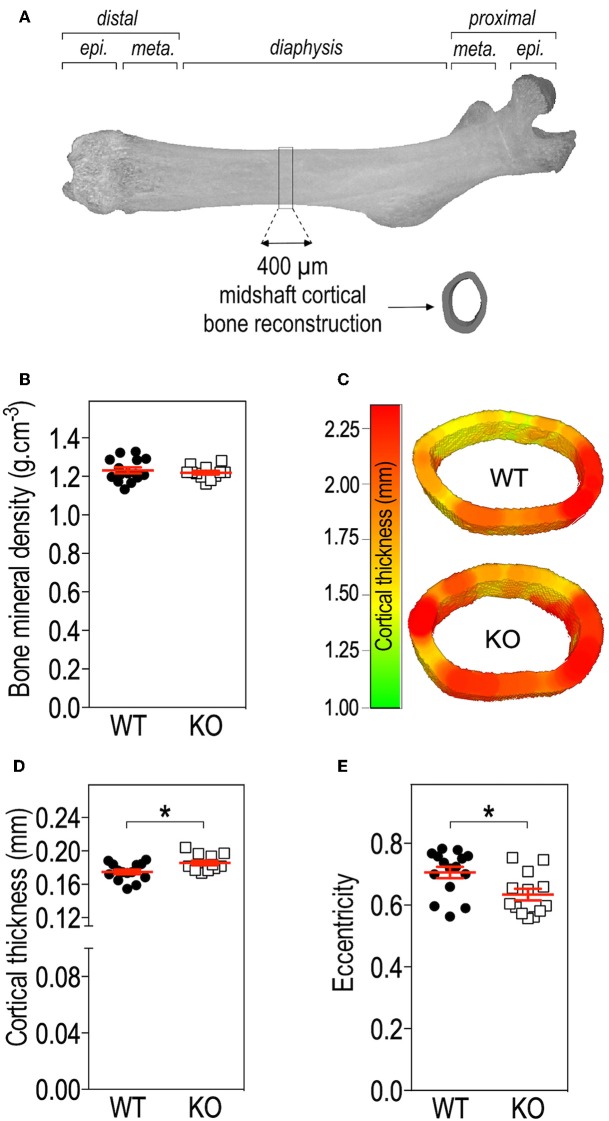
*Fmr1-*deficiency does not impact bone mineral density (BMD) but modifies cortical bone architecture. **(A)** Location of ROI used for mid-shaft cortical bone 3D reconstruction of a 400 μm diaphysis section. meta., metaphysis; epi., epiphysis. **(B)** Bone mineral density. **(C)** Representative 3D reconstructions of cortical thickness in *Fmr1*-KO and WT femurs. Average cortical thickness **(D)** and cortical bone eccentricity **(E)** in *Fmr1*-KO and WT 4 months-old male animals. For **(B,D,E)**, data are presented as dot-plots featuring means ± SEM in red. Statistical significance of differences was measured using 2-tailed Student's *T*-test **(D)** or Mann & Whitney *U*-test **(E)**. *p < 0.05. *n* = 12–15 animals/group.

To further resolve the microstructure of bone in *Fmr1*-WT and KO mice, we analyzed BMD and structure of femurs at the level of the mid-shaft cortical bone, using high resolution micro-computerized X-ray tomography (Figure 2A, Table 1). Volumetric BMD measured in the mid-shaft was not significantly affected by *Fmr1-*deficiency (Table 1, Figure 2B). We did not observe changes in the mean total crossectional object area, the mean object area per slice or the mean cortical area fraction (Table 1).

Qualitative analysis of transversal sections of mid-shaft cortical bone showed that *Fmr1-*deficiency impact the cortical pattern and skews toward higher ranges the cortical thickness distribution (Figure 2C). Quantitative analysis over a 400 μm mid-shaft block revealed that *Fmr1*-KO mice exhibit a significant increase in average cortical thickness as compared to WT (Table 1, Figure 2D). Furthermore, the mean polar MMI, an estimator of bone strength was not significantly impacted in *Fmr1*-KO mice (Table 1).

We also assessed cortical eccentricity, a measure of bone cross-sectional geometry, which is known to influence biomechanical response of the bone during habitual loading. Cortical eccentricity appeared significantly shifted toward lower values in *Fmr1*-KO femurs (Table 1, Figure 2E). This is indicative of a rounder shape of the bone at the mid-shaft level in *Fmr1-*KO animals as compared to WT.

**Figure 3:**
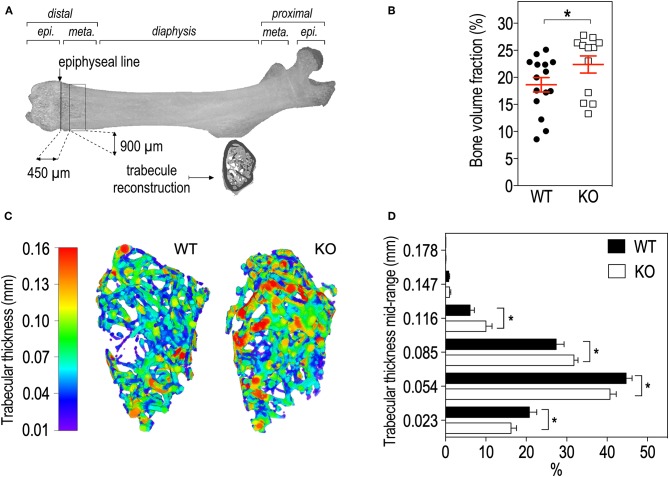
*Fmr1*-deficiency lowers trabecular bone volume and shifts the trabecular thickness distribution to higher values. **(A)** Location of ROI used for trabecular bone 3D reconstruction over a 900 μm bone segment located 450 μm distal to the epiphyseal line. meta., Metaphysis; epi., epiphysis. **(B)** Average bone volume fraction in *Fmr1*-KO and WT femurs. **(C)** Representative 3D reconstructions of trabecular thickness in *Fmr1*-KO and WT femurs. **(D)** Trabecular thickness distribution in reconstructed *Fmr1*-KO and WT ROI. Data are presented as means ± SEM. For **(B)**, statistical significance of differences was measured using 2-tailed Student's *T*-test: *p < 0.05). For **(D)** 2-way ANOVA: *p*(Genotype) = 0.3103, *p*(Range) < 0.0001, *p*(Interaction) = 0.0037; Fisher's LSD *post-hoc* tests for genotype-wise comparisons. *p < 0.05. *n* = 12–15 animals/group.

We then studied bone trabecular microstructure by analyzing transversal sections of a 900 μm segment located in the distal femoral metaphysis (Figure 3A). *Fmr1*-KO mice displayed a significant increase in the bone volume fraction as compared to WT littermates (Table 2, Figure 3B). The average trabecular numbers, average trabecular separation, and average trabecular thickness were not significantly impacted by *Fmr1*-deficiency (Table 2). However, when looking in closer details at the trabecular thickness distributions over the 900 μm segment, we observed that *Fmr1-*deficiency significantly shifted to higher ranges the trabecular thickness (Figure 3C), and this shift was significant (Figure 3D). Furthermore, the total volume of trabecular pores was significantly decreased in *Fmr1-*KO animals (Table 2), in line with the increase in bone volume fraction.

Finally, we measured circulating osteocalcin, a bone-hormone exclusively secreted by osteoblasts with roles in bone formation, metabolism, and adaptation to exercise (25). In *Fmr1*-KO animals, serum osteocalcin levels were similar to *Fmr1*-WT levels (Supplementary Figure 2).

### *Fmr1-*Deficiency Leads to Increased Physical Activity in Mouse

**Figure 4:**
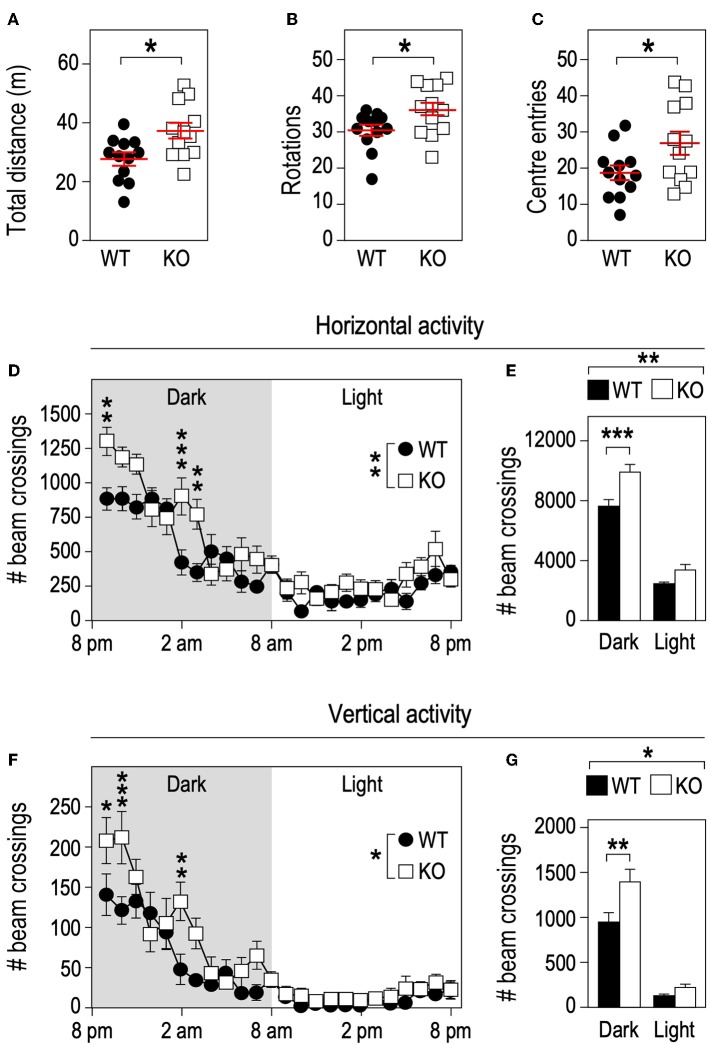
*Fmr1*-deficiency increases locomotor activity. *Fmr1*-KO and WT adult male littermates were monitored for 10 min in an open-field arena and total distance traveled **(A)**, number of rotations **(B)**, and center entries **(C)** were calculated. Horizontal activity measured in actimetry chambers over 24 h **(D)** and corresponding histograms of cumulative activity in dark/light phases **(E)**. Vertical activity measured in actimetry chambers over 24 h **(F)** and corresponding histograms of cumulative activity in dark/light phases **(G)**. *n* = 12 animals/group. For **(A–C)**, data are presented as dot-plots featuring means ± SEM. Statistical significance of differences was measured using 2-tailed Student's *T*-tests. *p < 0.05. For **(D–G)**, data are presented as means ± SEM. For **(D)** 2-way ANOVA: *p*(Genotype) = 0.0053, *p*(Time) < 0.0001, *p*(Interaction) = 0.0002; Holm-Šidák's *post-hoc* tests for genotype-wise comparisons. **p < 0.01; ***p < 0.001. For **(E)** 2-way ANOVA: *p*(Genotype) = 0.0024, *p*(Phase) < 0.0001, *p*(Interaction) = 0.0599; Holm-Šidák's *post-hoc* tests for genotype-wise comparisons. **p < 0.01; ***p < 0.0001. For **(F)** 2-way ANOVA: *p*(Genotype) = 0.0394, *p*(Phase) < 0.0001, *p*(Interaction) = 0.0006; Holm-Šidák's *post-hoc* tests for genotype-wise comparisons. *p < 0.05, **p < 0.01; ***p < 0.001. For **(G)** 2-way ANOVA: *p*(Genotype) = 0.0348, *p*(Phase) < 0.0001, *p*(Interaction) = 0.042; Holm-Šidák's *post-hoc* tests for genotype-wise comparisons. *p < 0.05, **p < 0.01.

Physical activity is a strong contributor to adaptations in bone architecture (26) and the changes we observed in *Fmr1-*KO mouse in terms of body composition and bone microstructure recapitulate the ones observed in mice subjected to exercise (27). We therefore monitored the impact of *Fmr1*-deficiency on spontaneous locomotor activity. In the open field test, *Fmr1*-KO mice were significantly more active than WT littermates in total distance traveled (Figure 4A), number of rotations (Figure 4B) and showed a higher number of center entries (Figure 4C). As open-field recordings are performed over a short period of time (10 min.), we then monitored continuous locomotor activity in *Fmr1-*KO and WT littermates in actimetry chambers over 24 h in a standard dark-light cycle. Independently of genotypes, horizontal (Figures 4D,E) and vertical activities (rearings, Figures 4F,G) display a characteristic nychthemeral rhythm, with two nocturnal peaks and reduced movements during the light phase. There was a significant effect of genotype over time on horizontal activity (Figure 4D). Cumulative horizontal activity was significantly increased in the nocturnal phase for *Fmr1-KO* animals as compared to their WT littermates (Figure 4E). Similarly, there was a significant effect of genotype over time on vertical activity (Figure 4F). Cumulative vertical activity was significantly increased in the nocturnal phase for *Fmr1-KO* animals as compared to their WT littermates (Figure 4G). These results indicate that *Fmr1*-deficient mice display a sustained hyperactivity as compared to wildtype mice.

## Discussion

In the present study, we show that *Fmr1*-deficiency in mice, as a model of FXS pathology, translates into increased body weight, skeleton length, and volume. Some studies report a general overgrowth in boys affected by FXS (13, 15) and increased stature and height (14), which is in line with our observations in *Fmr1-*KO mice. In *Fmr1-*KO mice, overweight and overgrowth are accompanied by an increase in muscular mass and a reduction of intra-abdominal adiposity. The later result corroborates our previous observations of reduced WAT, decreased circulating leptin and increased lipid catabolism in *Fmr1-*KO mice (11) and previous findings of reduced lipid storage in FXS *Drosophila* model (28). This suggests that *Fmr1-*deficiency provokes fat mass redistribution impacting visceral WAT and sparing subcutaneous WAT and BAT. Whole-body energy expenditure, that is critically regulated by BAT, was also not impacted by *Fmr1*-deficiency, as we have previously shown using indirect calorimetry (11). Also, body temperature of *Fmr1-*KO mice was similar to WT (Leboucher & Davidovic, unpublished observations). All these data confirm the lack of overt phenotype related to BAT in *Fmr1-*KO animals and suggest that this tissue is unlikely involved in the described phenotypes. No epidemiological study on the FXS population is available to-date regarding the adiposity in typical FXS patients. The reduced adiposity we observed in *Fmr1-*KO mice could at first sight be contradictory with the reports of hyperphagia and obesity in a rare subset of Fragile X patients displaying Prader Willi-like (PWL) phenotypes (29). However, these traits are not observed in typical FXS patients (11, 13, 30). Our results warrant further clinical studies to characterize body composition in FXS patients.

When looking closer at bone microstructure parameters, we observed that BMD was not significantly affected by *Fmr1-*deficiency, indicating that the bone mineralization process is unlikely affected in *Fmr1*-KO animals. However, bone architecture of *Fmr1*-KO mice is impacted, with decreased volume of trabecular pore, increased trabecular bone volume fraction and a shift toward higher trabecular thickness ranges, accompanied by increased cortical thickness, and eccentricity. The changes in bone microstructure which we highlight in *Fmr1*-KO animals resemble the ones observed in humans (31) or animals (27) subjected to long-term physical exercise. Indeed, physical activity is not only a strong contributor to adaptations in bone architecture but it also provokes body mass redistribution, notably a decrease in fat mass and an increase in the lean mass (26), which is what we observe in *Fmr1-*KO mice as compared to control mice. Also, the changes in shape of the proximal femur with an increased eccentricity could reflect the changing loading pattern during growth due to increased physical activity. Indeed, changes in bone morphology and strength can be enhanced through functional loading as shown in human studies (32, 33). As bone architecture reflects the history load originating both from muscle contractions as well as from the effect of gravity, the increased cortical thickness and the shift toward higher trabecular thickness in *Fmr1*-KO animals could correspond to a functional adaptation to the mechanical stress induced by increased physical activity and body weight. We therefore hypothesize that the increased physical activity observed in *Fmr1-*KO mice could indirectly participate to changes in body composition. Importantly, physical activity behavior is mostly centrally regulated and thus could be a possible consequence of *Fmr1-*deficiency in the CNS. Increased daily locomotor activity alterations had been previously well-documented in *Drosophila* FXS model (34) and in younger 8 weeks-old male *Fmr1*-KO animals (35). Our results indicate that 4-months old *Fmr1*-deficient mice display a sustained increase in physical activity as compared to wildtype mice, suggesting that hyperactivity-related phenotypes are not transient and persist in adulthood. This agrees with attention deficits and hyperactivity disorder (ADHD) being a frequent comorbidity diagnosed in FXS patients (13). Furthermore, physical activity is known to trigger beneficial metabolic changes, notably reduction in circulating triglycerides and cholesterol and better response to insulin, which are phenotypes we have recently described in the *Fmr1*-KO mice (11). All these data point toward important roles for *FMR1* gene at multiple levels in the organism: both central and peripheral.

FMRP, the protein encoded by the *FMR1* gene is a RNA-binding protein which controls mRNA translation. FMRP is widely expressed in the organism, including in adipose tissue, however, FMRP is absent from the adult muscle (8–12). It is therefore unlikely that FMRP will directly participate to muscle remodeling. In addition to increased physical activity, dysregulated translation regulation in bone tissue in the absence of FMRP could also participate to the skeletal and bone microstructure changes we observed here. Also, craniofacial abnormalities have been previously identified in both FXS patients and FXS mouse model (20), and this occurs independently of physical activity, suggesting that FMRP could have a direct role on bone formation/resorption processes. Further molecular studies would be required to identify FMRP mRNA targets in bone and its contribution to the shaping of bone microstructure.

Our study has some limitations. First, we have assessed bone phenotypes at a single age (4-month-old) and only in male animals. The stability of these phenotypes over time (during growth and aging) and the impact of sex should be further investigated. Circulating bone hormones, including osteocalcin levels, could also be assessed during growth. Second, detailed histo-morphometric studies of bone coupled to *in vitro* studies in osteoblasts and osteoclasts derived from *Fmr1*-KO and WT mice could contribute to identify the mechanism by which *Fmr1-*deficiency regulates bone morphology. Third, to estimate whether the changes in body composition are accompanied with improved physical capacities, *Fmr1*-KO mice could be subjected to forced exercise training in a treadmill combined with indirect calorimetry. Finally, further studies in FXS patients are warranted to assess the translationality of our findings in FXS mouse model. In this context, the use of Dual Energy X-Ray Absorptiometry (DEXA) technology, easily amenable both to mice, and humans to study visceral fat mass and lean body mass would enable an in-depth characterization of body mass distribution in FXS.

To our knowledge, this study is the first to document changes in body mass composition, skeleton, and bone architecture induced by *Fmr1-*deficiency in mice. These changes may result in part from adaptations to the hyperactivity and increased body weight that we highlight in *Fmr1-*KO mice or from the loss of the translational regulator FMRP in bone or adipose tissue. We have previously shown that *Fmr1*-KO mice present a variety of metabolic changes, notably increased glucose, and insulin tolerance as well as a metabolic shift toward increased lipid catabolism, this being likely mediated by the loss of FMRP-mediated hepatic translation repression (11). To which extend these metabolic abnormalities could also be linked to increased physical activity remains to be investigated. Given that recent studies have shown that a dynamic crosstalk between peripheral organs is required for the optimal control of metabolic homeostasis (36, 37), our study further paves the way for further investigation of skeletal and metabolic changes in FXS patients.

## Data Availability Statement

The datasets generated for this study are available on request to the corresponding author.

## Ethics Statement

All animal studies were conducted in facilities accredited by French legal authorities (Direction Départementale de Protection des Populations des Alpes-Maritimes, accreditation #C-06-152-5). Procedures involving animal experimentation were approved by the local animal experimentation ethics committee and the French Ministère de l'Enseignement Supérieur et de la Recherche (agreement #00788.01 and #05224.01).

## Author Contributions

LD designed the project and wrote the manuscript. AL, PB-M, DP, and LD carried out the experiments. AL, DP, and LD analyzed the data. XM provided advices for tomography acquisition and 3D reconstruction. AL performed 2D analyses and 3D modelisation. E-ZA interpreted the tomography data. AL, PB-M, E-ZA, XM, and DP revised the manuscript.

### Conflict of Interest

The authors declare that the research was conducted in the absence of any commercial or financial relationships that could be construed as a potential conflict of interest.
