# Supplementary material for: Fmr1-Deficiency Impacts Body Composition, Skeleton, and Bone Microstructure in a Mouse Model of Fragile X Syndrome
Source: Front Endocrinol (Lausanne). 2019 Oct 2;10:678. doi: 10.3389/fendo.2019.00678 (PMC6783488; doi:10.3389/fendo.2019.00678)
Supplement: Supplementary file 1 [file Data_Sheet_1.PDF]

Supplementary Material

Supplementary Table 1. Comparison analysis of ratios relative to body mass distribution and skeleton in *Fmr1*-KO and WT littermates (Relative to Figure 1). For calculations, *Tibialis anterior* and adipose tissue weights were in mg and body weight in g. All lengths were in mm. Mean and standard deviations (SD) are reported for *Fmr1*-WT and KO animals, as well as p-value for the Student's T-test. n=15-18 animals/group.

Ratio	Mean WT	SD WT	Mean KO	SD KO	p-value
***Tibialis anterior* weight / Body weight**	1.800	0.198	1.854	0.225	0.4858
**Femur length / Skeleton length**	0.188	0.005	0.188	0.004	0.7664
**L3-L6 length / Skeleton length**	0.147	0.003	0.149	0.003	0.0780

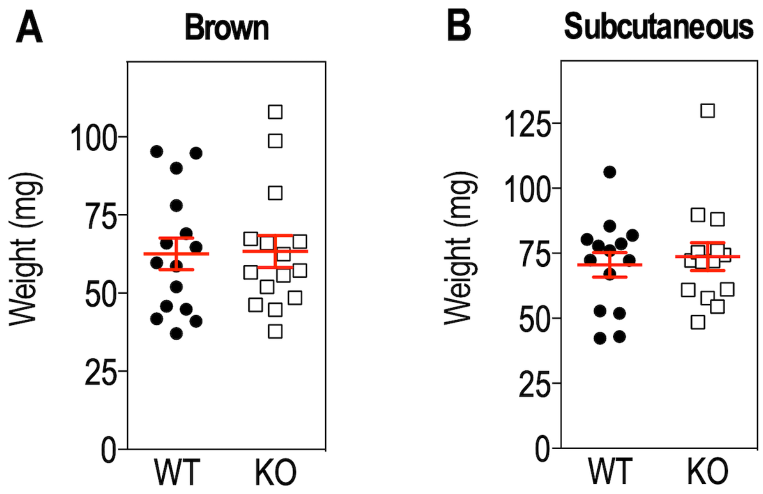

Supplementary Figure 1. *Fmr1*-deficiency does not impact brown or subcutaneous white adipose tissue weight (relative to Figure 1).

Weight of interscapular brown adipose tissue (*A*) and subcutaneous white adipose tissues (*B*) in 4 months-old *Fmr1*-WT and KO mice. Data are presented as dot-plots featuring means ± SEM in red. Student T-test p-value: 0.912 (*A*), 0.637 (*B*). n=13-15 animals/group.

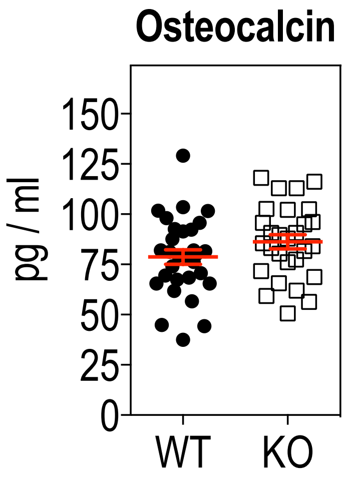

Supplementary Figure 2. *Fmr1*-deficiency does not impact the levels of circulating osteocalcin.

Serum levels of osteocalcin in 4 months-old *Fmr1*-WT and KO mice. Data are presented as dot-plots featuring means ± SEM in red. Student T-test p-value: 0.134. n=28-30 animals/group.
